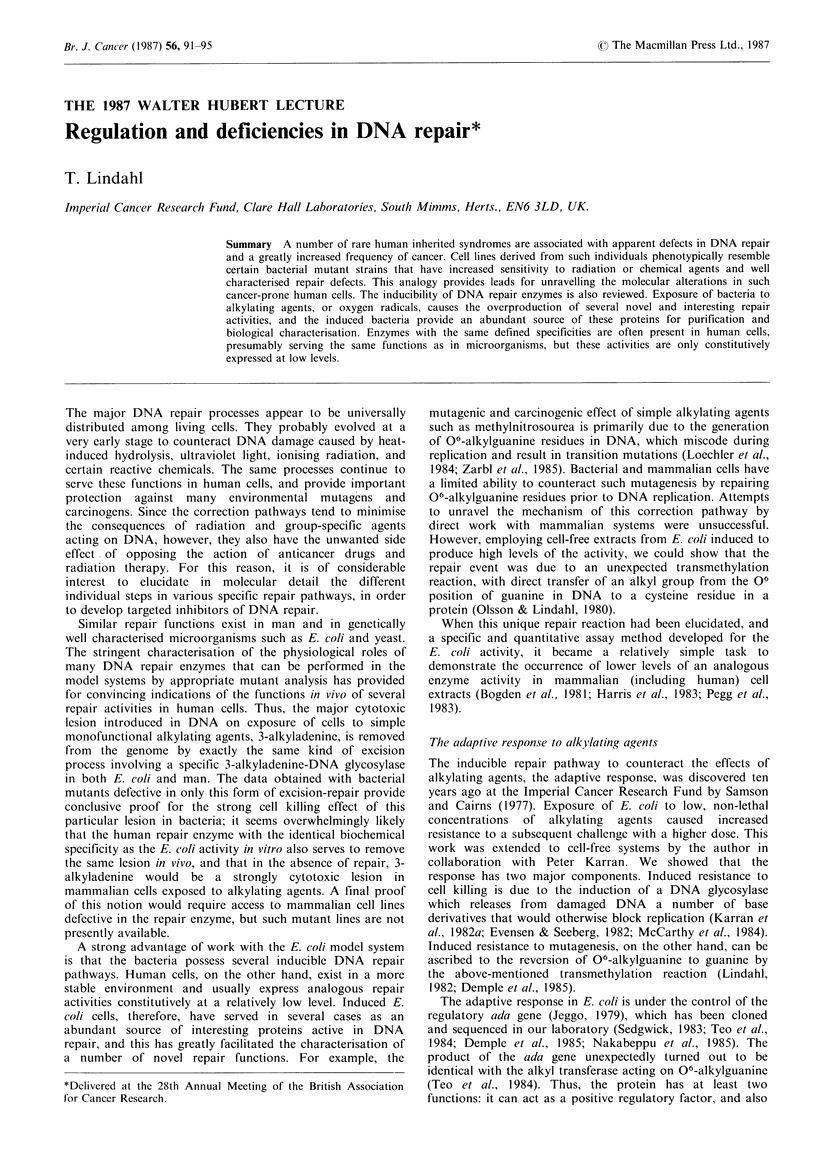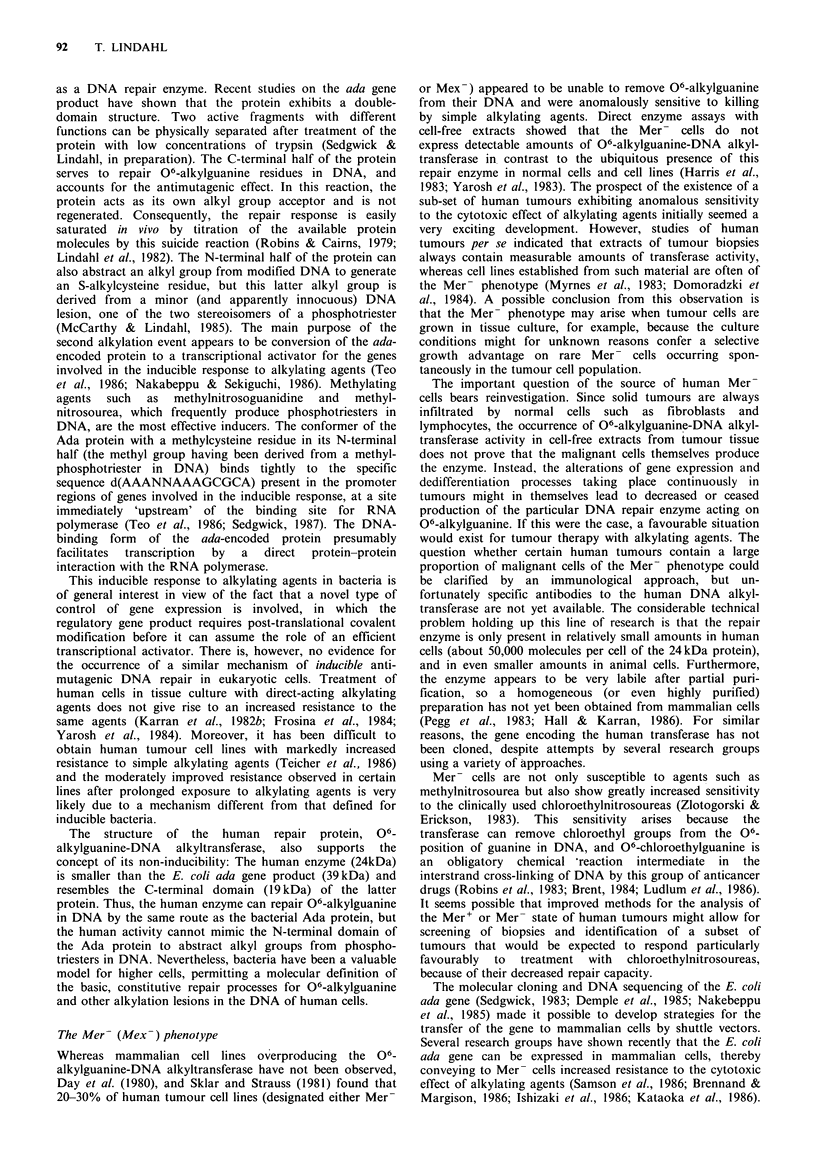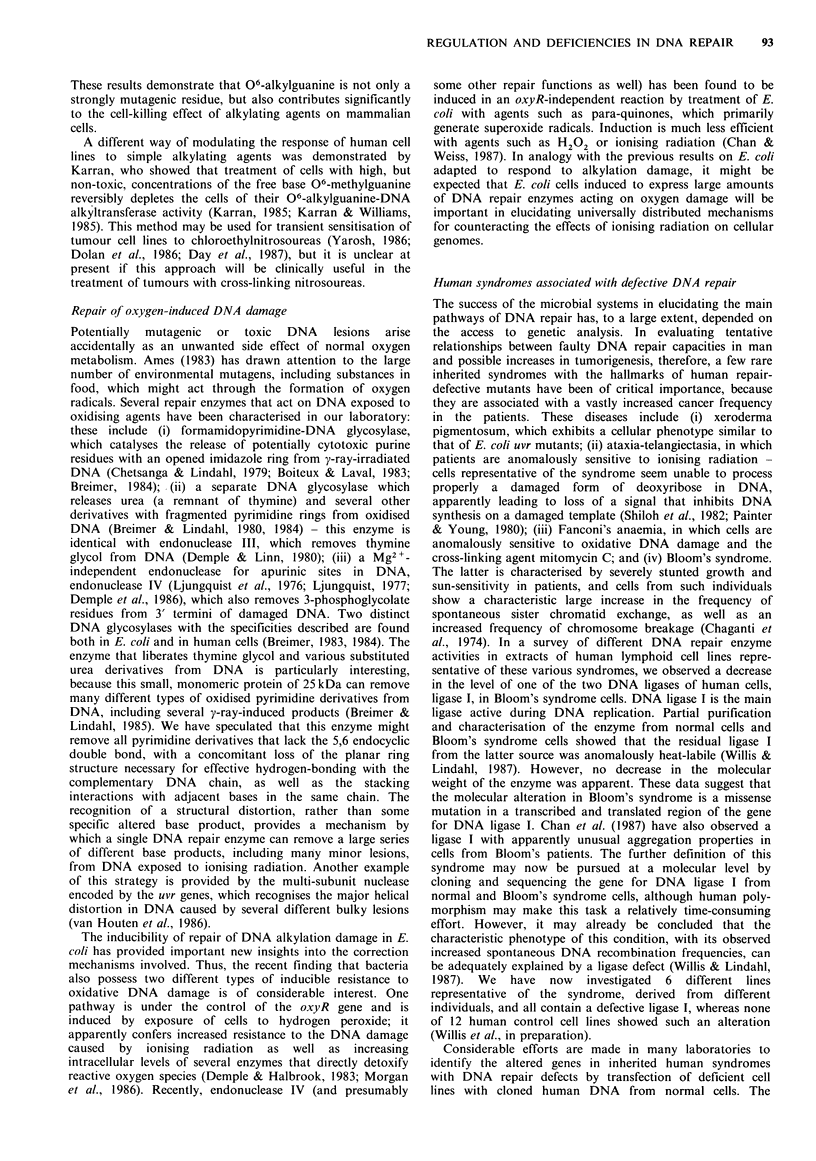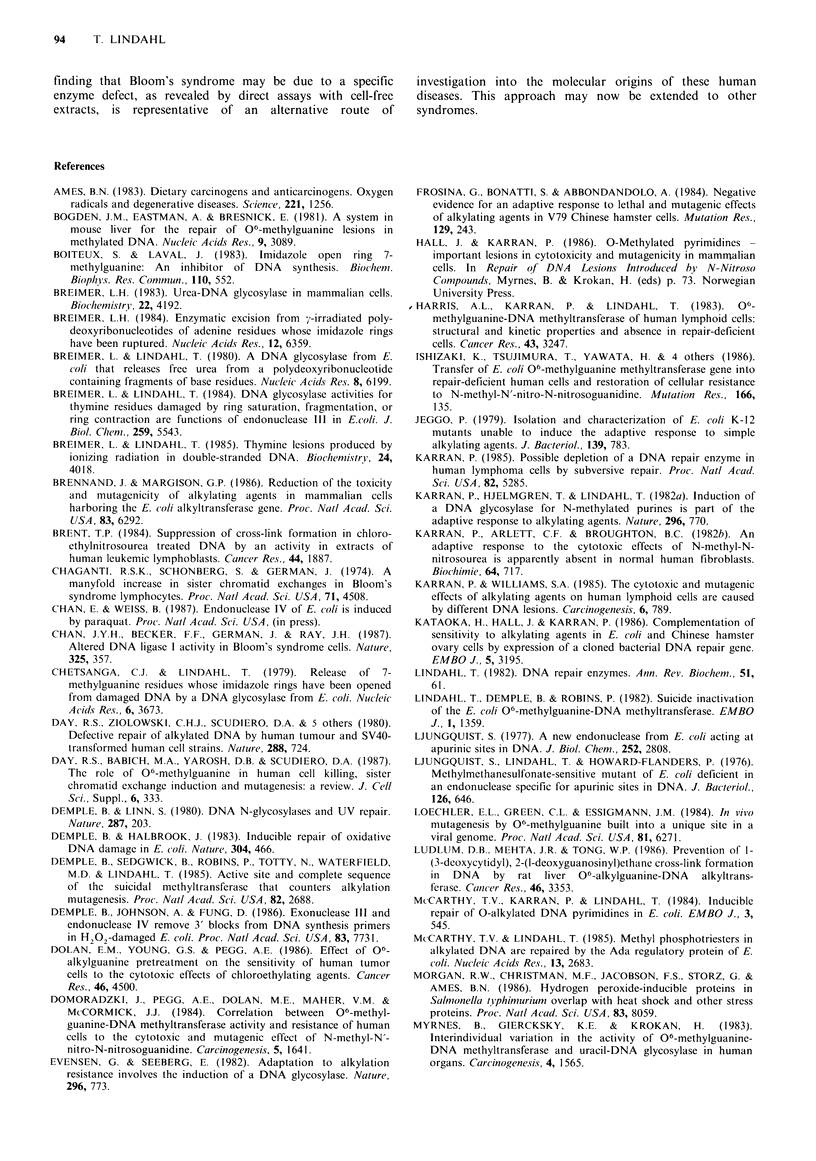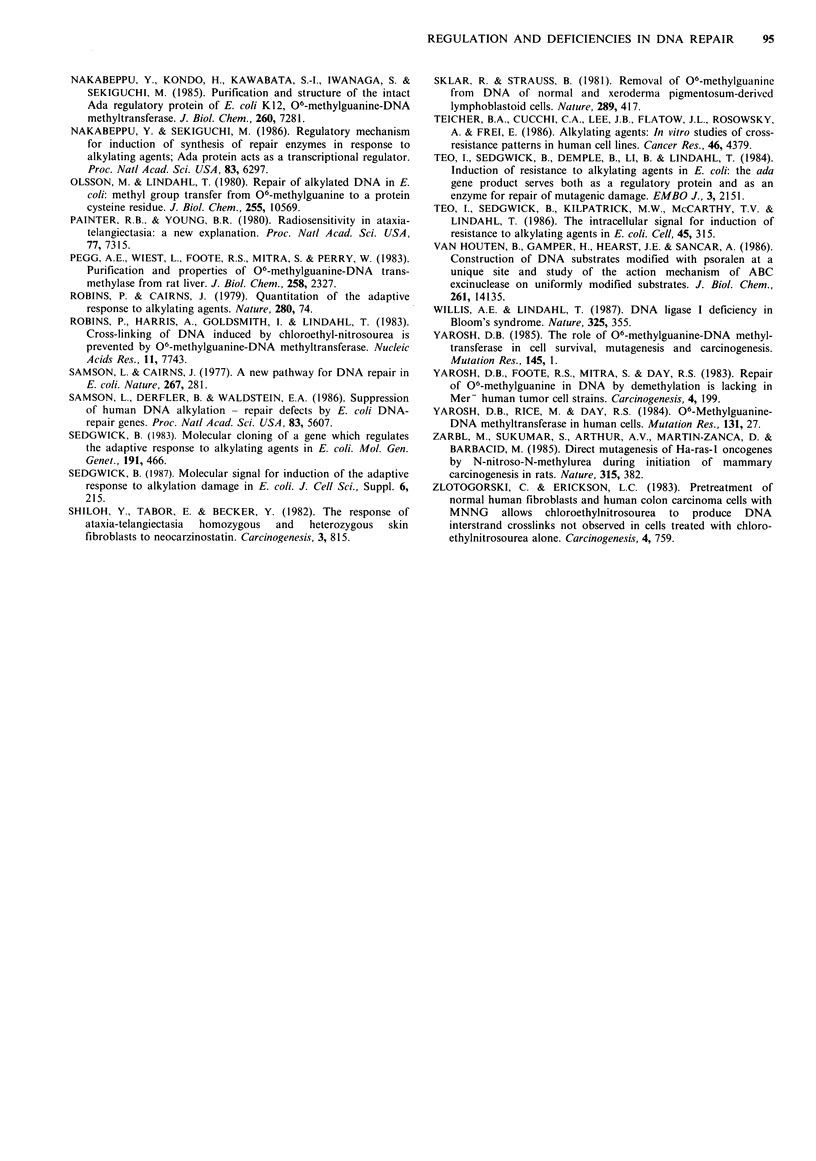# The 1987 Walter Hubert lecture. Regulation and deficiencies in DNA repair.

**DOI:** 10.1038/bjc.1987.163

**Published:** 1987-08

**Authors:** T. Lindahl

**Affiliations:** Imperial Cancer Research Fund, Clare Hall Laboratories, South Mimms, Herts., UK.

## Abstract

A number of rare human inherited syndromes are associated with apparent defects in DNA repair and a greatly increased frequency of cancer. Cell lines derived from such individuals phenotypically resemble certain bacterial mutant strains that have increased sensitivity to radiation or chemical agents and well characterised repair defects. This analogy provides leads for unravelling the molecular alterations in such cancer-prone human cells. The inducibility of DNA repair enzymes is also reviewed. Exposure of bacteria to alkylating agents, or oxygen radicals, causes the overproduction of several novel and interesting repair activities, and the induced bacteria provide an abundant source of these proteins for purification and biological characterisation. Enzymes with the same defined specificities are often present in human cells, presumably serving the same functions as in microorganisms, but these activities are only constitutively expressed at low levels.


*Br. J. Cancer* (1987) **56**, 91–95 © The Macmillan Press Ltd., 1987

THE 1987 WALTER HUBERT LECTURE

### Regulation and deficiencies in DNA repair*

T. Lindahl

*Imperial Cancer Research Fund, Clare Hall Laboratories, South Mimms, Herts., EN6 3LD, UK.*

**Summary** A number of rare human inherited syndromes are associated with apparent defects in DNA repair and a greatly increased frequency of cancer. Cell lines derived from such individuals phenotypically resemble certain bacterial mutant strains that have increased sensitivity to radiation or chemical agents and well characterised repair defects. This analogy provides leads for unravelling the molecular alterations in such cancer-prone human cells. The inducibility of DNA repair enzymes is also reviewed. Exposure of bacteria to alkylating agents, or oxygen radicals, causes the overproduction of several novel and interesting repair activities, and the induced bacteria provide an abundant source of these proteins for purification and biological characterisation. Enzymes with the same defined specificities are often present in human cells, presumably serving the same functions as in microorganisms, but these activities are only constitutively expressed at low levels.

The major DNA repair processes appear to be universally distributed among living cells. They probably evolved at a very early stage to counteract DNA damage caused by heat-induced hydrolysis, ultraviolet light, ionising radiation, and certain reactive chemicals. The same processes continue to serve these functions in human cells, and provide important protection against many environmental mutagens and carcinogens. Since the correction pathways tend to minimise the consequences of radiation and group-specific agents acting on DNA, however, they also have the unwanted side effect of opposing the action of anticancer drugs and radiation therapy. For this reason, it is of considerable interest to elucidate in molecular detail the different individual steps in various specific repair pathways, in order to develop targeted inhibitors of DNA repair.

Similar repair functions exist in man and in genetically well characterised microorganisms such as *E. coli* and yeast. The stringent characterisation of the physiological roles of many DNA repair enzymes that can be performed in the model systems by appropriate mutant analysis has provided for convincing indications of the functions *in vivo* of several repair activities in human cells. Thus, the major cytotoxic lesion introduced in DNA on exposure of cells to simple monofunctional alkylating agents, 3-alkyladenine, is removed from the genome by exactly the same kind of excision process involving a specific 3-alkyladenine-DNA glycosylase in both *E. coli* and man. The data obtained with bacterial mutants defective in only this form of excision-repair provide conclusive proof for the strong cell killing effect of this particular lesion in bacteria; it seems overwhelmingly likely that the human repair enzyme with the identical biochemical specificity as the *E. coli* activity *in vitro* also serves to remove the same lesion *in vivo*, and that in the absence of repair, 3-alkyladenine would be a strongly cytotoxic lesion in mammalian cells exposed to alkylating agents. A final proof of this notion would require access to mammalian cell lines defective in the repair enzyme, but such mutant lines are not presently available.

A strong advantage of work with the *E. coli* model system is that the bacteria possess several inducible DNA repair pathways. Human cells, on the other hand, exist in a more stable environment and usually express analogous repair activities constitutively at a relatively low level. Induced *E. coli* cells, therefore, have served in several cases as an abundant source of interesting proteins active in DNA repair, and this has greatly facilitated the characterisation of a number of novel repair functions. For example, the mutagenic and carcinogenic effect of simple alkylating agents such as methylnitrosourea is primarily due to the generation of $O^6$-alkylguanine residues in DNA, which miscode during replication and result in transition mutations (Loechler *et al.*, 1984; Zarbl *et al.*, 1985). Bacterial and mammalian cells have a limited ability to counteract such mutagenesis by repairing $O^6$-alkylguanine residues prior to DNA replication. Attempts to unravel the mechanism of this correction pathway by direct work with mammalian systems were unsuccessful. However, employing cell-free extracts from *E. coli* induced to produce high levels of the activity, we could show that the repair event was due to an unexpected transmethylation reaction, with direct transfer of an alkyl group from the $O^6$ position of guanine in DNA to a cysteine residue in a protein (Olsson & Lindahl, 1980).

When this unique repair reaction had been elucidated, and a specific and quantitative assay method developed for the *E. coli* activity, it became a relatively simple task to demonstrate the occurrence of lower levels of an analogous enzyme activity in mammalian (including human) cell extracts (Bogden *et al.*, 1981; Harris *et al.*, 1983; Pegg *et al.*, 1983).

### *The adaptive response to alkylating agents*

The inducible repair pathway to counteract the effects of alkylating agents, the adaptive response, was discovered ten years ago at the Imperial Cancer Research Fund by Samson and Cairns (1977). Exposure of *E. coli* to low, non-lethal concentrations of alkylating agents caused increased resistance to a subsequent challenge with a higher dose. This work was extended to cell-free systems by the author in collaboration with Peter Karran. We showed that the response has two major components. Induced resistance to cell killing is due to the induction of a DNA glycosylase which releases from damaged DNA a number of base derivatives that would otherwise block replication (Karran *et al.*, 1982*a*; Evensen & Seeberg, 1982; McCarthy *et al.*, 1984). Induced resistance to mutagenesis, on the other hand, can be ascribed to the reversion of $O^6$-alkylguanine to guanine by the above-mentioned transmethylation reaction (Lindahl, 1982; Demple *et al.*, 1985).

The adaptive response in *E. coli* is under the control of the regulatory *ada* gene (Jeggo, 1979), which has been cloned and sequenced in our laboratory (Sedgwick, 1983; Teo *et al.*, 1984; Demple *et al.*, 1985; Nakabeppu *et al.*, 1985). The product of the *ada* gene unexpectedly turned out to be identical with the alkyl transferase acting on $O^6$-alkylguanine (Teo *et al.*, 1984). Thus, the protein has at least two functions: it can act as a positive regulatory factor, and also

*Delivered at the 28th Annual Meeting of the British Association for Cancer Research.

as a DNA repair enzyme. Recent studies on the *ada* gene product have shown that the protein exhibits a double-domain structure. Two active fragments with different functions can be physically separated after treatment of the protein with low concentrations of trypsin (Sedgwick & Lindahl, in preparation). The C-terminal half of the protein serves to repair $O^6$-alkylguanine residues in DNA, and accounts for the antimutagenic effect. In this reaction, the protein acts as its own alkyl group acceptor and is not regenerated. Consequently, the repair response is easily saturated *in vivo* by titration of the available protein molecules by this suicide reaction (Robins & Cairns, 1979; Lindahl *et al.*, 1982). The N-terminal half of the protein can also abstract an alkyl group from modified DNA to generate an S-alkylcysteine residue, but this latter alkyl group is derived from a minor (and apparently innocuous) DNA lesion, one of the two stereoisomers of a phosphotriester (McCarthy & Lindahl, 1985). The main purpose of the second alkylation event appears to be conversion of the *ada*-encoded protein to a transcriptional activator for the genes involved in the inducible response to alkylating agents (Teo *et al.*, 1986; Nakabeppu & Sekiguchi, 1986). Methylating agents such as methylnitrosoguanidine and methylnitrosourea, which frequently produce phosphotriesters in DNA, are the most effective inducers. The conformer of the Ada protein with a methylcysteine residue in its N-terminal half (the methyl group having been derived from a methylphosphotriester in DNA) binds tightly to the specific sequence d(AAANNAAAGCGCA) present in the promoter regions of genes involved in the inducible response, at a site immediately 'upstream' of the binding site for RNA polymerase (Teo *et al.*, 1986; Sedgwick, 1987). The DNA-binding form of the *ada*-encoded protein presumably facilitates transcription by a direct protein–protein interaction with the RNA polymerase.

This inducible response to alkylating agents in bacteria is of general interest in view of the fact that a novel type of control of gene expression is involved, in which the regulatory gene product requires post-translational covalent modification before it can assume the role of an efficient transcriptional activator. There is, however, no evidence for the occurrence of a similar mechanism of *inducible* antimutagenic DNA repair in eukaryotic cells. Treatment of human cells in tissue culture with direct-acting alkylating agents does not give rise to an increased resistance to the same agents (Karran *et al.*, 1982*b*; Frosina *et al.*, 1984; Yarosh *et al.*, 1984). Moreover, it has been difficult to obtain human tumour cell lines with markedly increased resistance to simple alkylating agents (Teicher *et al.*, 1986) and the moderately improved resistance observed in certain lines after prolonged exposure to alkylating agents is very likely due to a mechanism different from that defined for inducible bacteria.

The structure of the human repair protein, $O^6$-alkylguanine-DNA alkyltransferase, also supports the concept of its non-inducibility: The human enzyme (24kDa) is smaller than the *E. coli ada* gene product (39 kDa) and resembles the C-terminal domain (19 kDa) of the latter protein. Thus, the human enzyme can repair $O^6$-alkylguanine in DNA by the same route as the bacterial Ada protein, but the human activity cannot mimic the N-terminal domain of the Ada protein to abstract alkyl groups from phosphotriesters in DNA. Nevertheless, bacteria have been a valuable model for higher cells, permitting a molecular definition of the basic, constitutive repair processes for $O^6$-alkylguanine and other alkylation lesions in the DNA of human cells.

### *The Mer$^-$ (Mex$^-$) phenotype*

Whereas mammalian cell lines overproducing the $O^6$-alkylguanine-DNA alkyltransferase have not been observed, Day *et al.* (1980), and Sklar and Strauss (1981) found that 20–30% of human tumour cell lines (designated either Mer$^-$ or Mex$^-$) appeared to be unable to remove $O^6$-alkylguanine from their DNA and were anomalously sensitive to killing by simple alkylating agents. Direct enzyme assays with cell-free extracts showed that the Mer$^-$ cells do not express detectable amounts of $O^6$-alkylguanine-DNA alkyltransferase in contrast to the ubiquitous presence of this repair enzyme in normal cells and cell lines (Harris *et al.*, 1983; Yarosh *et al.*, 1983). The prospect of the existence of a sub-set of human tumours exhibiting anomalous sensitivity to the cytotoxic effect of alkylating agents initially seemed a very exciting development. However, studies of human tumours *per se* indicated that extracts of tumour biopsies always contain measurable amounts of transferase activity, whereas cell lines established from such material are often of the Mer$^-$ phenotype (Myrnes *et al.*, 1983; Domoradzki *et al.*, 1984). A possible conclusion from this observation is that the Mer$^-$ phenotype may arise when tumour cells are grown in tissue culture, for example, because the culture conditions might for unknown reasons confer a selective growth advantage on rare Mer$^-$ cells occurring spontaneously in the tumour cell population.

The important question of the source of human Mer$^-$ cells bears reinvestigation. Since solid tumours are always infiltrated by normal cells such as fibroblasts and lymphocytes, the occurrence of $O^6$-alkylguanine-DNA alkyltransferase activity in cell-free extracts from tumour tissue does not prove that the malignant cells themselves produce the enzyme. Instead, the alterations of gene expression and dedifferentiation processes taking place continuously in tumours might in themselves lead to decreased or ceased production of the particular DNA repair enzyme acting on $O^6$-alkylguanine. If this were the case, a favourable situation would exist for tumour therapy with alkylating agents. The question whether certain human tumours contain a large proportion of malignant cells of the Mer$^-$ phenotype could be clarified by an immunological approach, but unfortunately specific antibodies to the human DNA alkyltransferase are not yet available. The considerable technical problem holding up this line of research is that the repair enzyme is only present in relatively small amounts in human cells (about 50,000 molecules per cell of the 24 kDa protein), and in even smaller amounts in animal cells. Furthermore, the enzyme appears to be very labile after partial purification, so a homogeneous (or even highly purified) preparation has not yet been obtained from mammalian cells (Pegg *et al.*, 1983; Hall & Karran, 1986). For similar reasons, the gene encoding the human transferase has not been cloned, despite attempts by several research groups using a variety of approaches.

Mer$^-$ cells are not only susceptible to agents such as methylnitrosourea but also show greatly increased sensitivity to the clinically used chloroethylnitrosoureas (Zlotogorski & Erickson, 1983). This sensitivity arises because the transferase can remove chloroethyl groups from the $O^6$-position of guanine in DNA, and $O^6$-chloroethylguanine is an obligatory chemical reaction intermediate in the interstrand cross-linking of DNA by this group of anticancer drugs (Robins *et al.*, 1983; Brent, 1984; Ludlum *et al.*, 1986). It seems possible that improved methods for the analysis of the Mer$^+$ or Mer$^-$ state of human tumours might allow for screening of biopsies and identification of a subset of tumours that would be expected to respond particularly favourably to treatment with chloroethylnitrosoureas, because of their decreased repair capacity.

The molecular cloning and DNA sequencing of the *E. coli ada* gene (Sedgwick, 1983; Demple *et al.*, 1985; Nakebeppu *et al.*, 1985) made it possible to develop strategies for the transfer of the gene to mammalian cells by shuttle vectors. Several research groups have shown recently that the *E. coli ada* gene can be expressed in mammalian cells, thereby conveying to Mer$^-$ cells increased resistance to the cytotoxic effect of alkylating agents (Samson *et al.*, 1986; Brennand & Margison, 1986; Ishizaki *et al.*, 1986; Kataoka *et al.*, 1986).

These results demonstrate that $O^6$-alkylguanine is not only a strongly mutagenic residue, but also contributes significantly to the cell-killing effect of alkylating agents on mammalian cells.

A different way of modulating the response of human cell lines to simple alkylating agents was demonstrated by Karran, who showed that treatment of cells with high, but non-toxic, concentrations of the free base $O^6$-methylguanine reversibly depletes the cells of their $O^6$-alkylguanine-DNA alkyltransferase activity (Karran, 1985; Karran & Williams, 1985). This method may be used for transient sensitisation of tumour cell lines to chloroethylnitrosoureas (Yarosh, 1986; Dolan *et al.*, 1986; Day *et al.*, 1987), but it is unclear at present if this approach will be clinically useful in the treatment of tumours with cross-linking nitrosoureas.

*Repair of oxygen-induced DNA damage*

Potentially mutagenic or toxic DNA lesions arise accidentally as an unwanted side effect of normal oxygen metabolism. Ames (1983) has drawn attention to the large number of environmental mutagens, including substances in food, which might act through the formation of oxygen radicals. Several repair enzymes that act on DNA exposed to oxidising agents have been characterised in our laboratory: these include (i) formamidopyrimidine-DNA glycosylase, which catalyses the release of potentially cytotoxic purine residues with an opened imidazole ring from $\gamma$-ray-irradiated DNA (Chetsanga & Lindahl, 1979; Boiteux & Laval, 1983; Breimer, 1984); (ii) a separate DNA glycosylase which releases urea (a remnant of thymine) and several other derivatives with fragmented pyrimidine rings from oxidised DNA (Breimer & Lindahl, 1980, 1984) – this enzyme is identical with endonuclease III, which removes thymine glycol from DNA (Demple & Linn, 1980); (iii) a $Mg^{2+}$-independent endonuclease for apurinic sites in DNA, endonuclease IV (Ljungquist *et al.*, 1976; Ljungquist, 1977; Demple *et al.*, 1986), which also removes 3-phosphoglycolate residues from 3′ termini of damaged DNA. Two distinct DNA glycosylases with the specificities described are found both in *E. coli* and in human cells (Breimer, 1983, 1984). The enzyme that liberates thymine glycol and various substituted urea derivatives from DNA is particularly interesting, because this small, monomeric protein of 25 kDa can remove many different types of oxidised pyrimidine derivatives from DNA, including several $\gamma$-ray-induced products (Breimer & Lindahl, 1985). We have speculated that this enzyme might remove all pyrimidine derivatives that lack the 5,6 endocyclic double bond, with a concomitant loss of the planar ring structure necessary for effective hydrogen-bonding with the complementary DNA chain, as well as the stacking interactions with adjacent bases in the same chain. The recognition of a structural distortion, rather than some specific altered base product, provides a mechanism by which a single DNA repair enzyme can remove a large series of different base products, including many minor lesions, from DNA exposed to ionising radiation. Another example of this strategy is provided by the multi-subunit nuclease encoded by the *uvr* genes, which recognises the major helical distortion in DNA caused by several different bulky lesions (van Houten *et al.*, 1986).

The inducibility of repair of DNA alkylation damage in *E. coli* has provided important new insights into the correction mechanisms involved. Thus, the recent finding that bacteria also possess two different types of inducible resistance to oxidative DNA damage is of considerable interest. One pathway is under the control of the *oxyR* gene and is induced by exposure of cells to hydrogen peroxide; it apparently confers increased resistance to the DNA damage caused by ionising radiation as well as increasing intracellular levels of several enzymes that directly detoxify reactive oxygen species (Demple & Halbrook, 1983; Morgan *et al.*, 1986). Recently, endonuclease IV (and presumably some other repair functions as well) has been found to be induced in an *oxyR*-independent reaction by treatment of *E. coli* with agents such as para-quinones, which primarily generate superoxide radicals. Induction is much less efficient with agents such as $H_2O_2$ or ionising radiation (Chan & Weiss, 1987). In analogy with the previous results on *E. coli* adapted to respond to alkylation damage, it might be expected that *E. coli* cells induced to express large amounts of DNA repair enzymes acting on oxygen damage will be important in elucidating universally distributed mechanisms for counteracting the effects of ionising radiation on cellular genomes.

*Human syndromes associated with defective DNA repair*

The success of the microbial systems in elucidating the main pathways of DNA repair has, to a large extent, depended on the access to genetic analysis. In evaluating tentative relationships between faulty DNA repair capacities in man and possible increases in tumorigenesis, therefore, a few rare inherited syndromes with the hallmarks of human repair-defective mutants have been of critical importance, because they are associated with a vastly increased cancer frequency in the patients. These diseases include (i) xeroderma pigmentosum, which exhibits a cellular phenotype similar to that of *E. coli uvr* mutants; (ii) ataxia-telangiectasia, in which patients are anomalously sensitive to ionising radiation – cells representative of the syndrome seem unable to process properly a damaged form of deoxyribose in DNA, apparently leading to loss of a signal that inhibits DNA synthesis on a damaged template (Shiloh *et al.*, 1982; Painter & Young, 1980); (iii) Fanconi's anaemia, in which cells are anomalously sensitive to oxidative DNA damage and the cross-linking agent mitomycin C; and (iv) Bloom's syndrome. The latter is characterised by severely stunted growth and sun-sensitivity in patients, and cells from such individuals show a characteristic large increase in the frequency of spontaneous sister chromatid exchange, as well as an increased frequency of chromosome breakage (Chaganti *et al.*, 1974). In a survey of different DNA repair enzyme activities in extracts of human lymphoid cell lines representative of these various syndromes, we observed a decrease in the level of one of the two DNA ligases of human cells, ligase I, in Bloom's syndrome cells. DNA ligase I is the main ligase active during DNA replication. Partial purification and characterisation of the enzyme from normal cells and Bloom's syndrome cells showed that the residual ligase I from the latter source was anomalously heat-labile (Willis & Lindahl, 1987). However, no decrease in the molecular weight of the enzyme was apparent. These data suggest that the molecular alteration in Bloom's syndrome is a missense mutation in a transcribed and translated region of the gene for DNA ligase I. Chan *et al.* (1987) have also observed a ligase I with apparently unusual aggregation properties in cells from Bloom's patients. The further definition of this syndrome may now be pursued at a molecular level by cloning and sequencing the gene for DNA ligase I from normal and Bloom's syndrome cells, although human polymorphism may make this task a relatively time-consuming effort. However, it may already be concluded that the characteristic phenotype of this condition, with its observed increased spontaneous DNA recombination frequencies, can be adequately explained by a ligase defect (Willis & Lindahl, 1987). We have now investigated 6 different lines representative of the syndrome, derived from different individuals, and all contain a defective ligase I, whereas none of 12 human control cell lines showed such an alteration (Willis *et al.*, in preparation).

Considerable efforts are made in many laboratories to identify the altered genes in inherited human syndromes with DNA repair defects by transfection of deficient cell lines with cloned human DNA from normal cells. The finding that Bloom's syndrome may be due to a specific enzyme defect, as revealed by direct assays with cell-free extracts, is representative of an alternative route of investigation into the molecular origins of these human diseases. This approach may now be extended to other syndromes.

**References**

AMES, B.N. (1983). Dietary carcinogens and anticarcinogens. Oxygen radicals and degenerative diseases. *Science*, **221**, 1256.

BOGDEN, J.M., EASTMAN, A. & BRESNICK, E. (1981). A system in mouse liver for the repair of O$^6$-methylguanine lesions in methylated DNA. *Nucleic Acids Res.*, **9**, 3089.

BOITEUX, S. & LAVAL, J. (1983). Imidazole open ring 7-methylguanine: An inhibitor of DNA synthesis. *Biochem. Biophys. Res. Commun.*, **110**, 552.

BREIMER, L.H. (1983). Urea-DNA glycosylase in mammalian cells. *Biochemistry*, **22**, 4192.

BREIMER, L.H. (1984). Enzymatic excision from $\gamma$-irradiated polydeoxyribonucleotides of adenine residues whose imidazole rings have been ruptured. *Nucleic Acids Res.*, **12**, 6359.

BREIMER, L. & LINDAHL, T. (1980). A DNA glycosylase from *E. coli* that releases free urea from a polydeoxyribonucleotide containing fragments of base residues. *Nucleic Acids Res.* **8**, 6199.

BREIMER, L. & LINDAHL, T. (1984). DNA glycosylase activities for thymine residues damaged by ring saturation, fragmentation, or ring contraction are functions of endonuclease III in *E.coli*. *J. Biol. Chem.*, **259**, 5543.

BREIMER, L. & LINDAHL, T. (1985). Thymine lesions produced by ionizing radiation in double-stranded DNA. *Biochemistry*, **24**, 4018.

BRENNAND, J. & MARGISON, G.P. (1986). Reduction of the toxicity and mutagenicity of alkylating agents in mammalian cells harboring the *E. coli* alkyltransferase gene. *Proc. Natl Acad. Sci. USA*, **83**, 6292.

BRENT, T.P. (1984). Suppression of cross-link formation in chloroethylnitrosourea treated DNA by an activity in extracts of human leukemic lymphoblasts. *Cancer Res.*, **44**, 1887.

CHAGANTI, R.S.K., SCHONBERG, S. & GERMAN, J. (1974). A manyfold increase in sister chromatid exchanges in Bloom's syndrome lymphocytes. *Proc. Natl Acad. Sci. USA*, **71**, 4508.

CHAN, E. & WEISS, B. (1987). Endonuclease IV of *E. coli* is induced by paraquat. *Proc. Natl Acad. Sci. USA*, (in press).

CHAN, J.Y.H., BECKER, F.F., GERMAN, J. & RAY, J.H. (1987). Altered DNA ligase I activity in Bloom's syndrome cells. *Nature*, **325**, 357.

CHETSANGA, C.J. & LINDAHL, T. (1979). Release of 7-methylguanine residues whose imidazole rings have been opened from damaged DNA by a DNA glycosylase from *E. coli*. *Nucleic Acids Res.*, **6**, 3673.

DAY, R.S., ZIOLOWSKI, C.H.J., SCUDIERO, D.A. & 5 others (1980). Defective repair of alkylated DNA by human tumour and SV40-transformed human cell strains. *Nature*, **288**, 724.

DAY, R.S., BABICH, M.A., YAROSH, D.B. & SCUDIERO, D.A. (1987). The role of O$^6$-methylguanine in human cell killing, sister chromatid exchange induction and mutagenesis: a review. *J. Cell Sci.*, Suppl., **6**, 333.

DEMPLE, B. & LINN, S. (1980). DNA N-glycosylases and UV repair. *Nature*, **287**, 203.

DEMPLE, B. & HALBROOK, J. (1983). Inducible repair of oxidative DNA damage in *E. coli*. *Nature*, **304**, 466.

DEMPLE, B., SEDGWICK, B., ROBINS, P., TOTTY, N., WATERFIELD, M.D. & LINDAHL, T. (1985). Active site and complete sequence of the suicidal methyltransferase that counters alkylation mutagenesis. *Proc. Natl Acad. Sci. USA*, **82**, 2688.

DEMPLE, B., JOHNSON, A. & FUNG, D. (1986). Exonuclease III and endonuclease IV remove 3′ blocks from DNA synthesis primers in $H_2O_2$-damaged *E. coli*. *Proc. Natl Acad. Sci. USA*, **83**, 7731.

DOLAN, E.M., YOUNG, G.S. & PEGG, A.E. (1986). Effect of O$^6$-alkylguanine pretreatment on the sensitivity of human tumor cells to the cytotoxic effects of chloroethylating agents. *Cancer Res.*, **46**, 4500.

DOMORADZKI, J., PEGG, A.E., DOLAN, M.E., MAHER, V.M. & McCORMICK, J.J. (1984). Correlation between O$^6$-methylguanine-DNA methyltransferase activity and resistance of human cells to the cytotoxic and mutagenic effect of N-methyl-N′-nitro-N-nitrosoguanidine. *Carcinogenesis*, **5**, 1641.

EVENSEN, G. & SEEBERG, E. (1982). Adaptation to alkylation resistance involves the induction of a DNA glycosylase. *Nature*, **296**, 773.

FROSINA, G., BONATTI, S. & ABBONDANDOLO, A. (1984). Negative evidence for an adaptive response to lethal and mutagenic effects of alkylating agents in V79 Chinese hamster cells. *Mutation Res.*, **129**, 243.

HALL, J. & KARRAN, P. (1986). O-Methylated pyrimidines – important lesions in cytotoxicity and mutagenicity in mammalian cells. In *Repair of DNA Lesions Introduced by N-Nitroso Compounds*, Myrnes, B. & Krokan, H. (eds) p. 73. Norwegian University Press.

HARRIS, A.L., KARRAN, P. & LINDAHL, T. (1983). O$^6$-methylguanine-DNA methyltransferase of human lymphoid cells: structural and kinetic properties and absence in repair-deficient cells. *Cancer Res.*, **43**, 3247.

ISHIZAKI, K., TSUJIMURA, T., YAWATA, H. & 4 others (1986). Transfer of *E. coli* O$^6$-methylguanine methyltransferase gene into repair-deficient human cells and restoration of cellular resistance to N-methyl-N′-nitro-N-nitrosoguanidine. *Mutation Res.*, **166**, 135.

JEGGO, P. (1979). Isolation and characterization of *E. coli* K-12 mutants unable to induce the adaptive response to simple alkylating agents. *J. Bacteriol.*, **139**, 783.

KARRAN, P. (1985). Possible depletion of a DNA repair enzyme in human lymphoma cells by subversive repair. *Proc. Natl Acad. Sci. USA*, **82**, 5285.

KARRAN, P., HJELMGREN, T. & LINDAHL, T. (1982*a*). Induction of a DNA glycosylase for N-methylated purines is part of the adaptive response to alkylating agents. *Nature*, **296**, 770.

KARRAN, P., ARLETT, C.F. & BROUGHTON, B.C. (1982*b*). An adaptive response to the cytotoxic effects of N-methyl-N-nitrosourea is apparently absent in normal human fibroblasts. *Biochimie*, **64**, 717.

KARRAN, P. & WILLIAMS, S.A. (1985). The cytotoxic and mutagenic effects of alkylating agents on human lymphoid cells are caused by different DNA lesions. *Carcinogenesis*, **6**, 789.

KATAOKA, H., HALL, J. & KARRAN, P. (1986). Complementation of sensitivity to alkylating agents in *E. coli* and Chinese hamster ovary cells by expression of a cloned bacterial DNA repair gene. *EMBO J.*, **5**, 3195.

LINDAHL, T. (1982). DNA repair enzymes. *Ann. Rev. Biochem.*, **51**, 61.

LINDAHL, T., DEMPLE, B. & ROBINS, P. (1982). Suicide inactivation of the *E. coli* O$^6$-methylguanine-DNA methyltransferase. *EMBO J.*, **1**, 1359.

LJUNGQUIST, S. (1977). A new endonuclease from *E. coli* acting at apurinic sites in DNA. *J. Biol. Chem.*, **252**, 2808.

LJUNGQUIST, S., LINDAHL, T. & HOWARD-FLANDERS, P. (1976). Methylmethanesulfonate-sensitive mutant of *E. coli* deficient in an endonuclease specific for apurinic sites in DNA. *J. Bacteriol.*, **126**, 646.

LOECHLER, E.L., GREEN, C.L. & ESSIGMANN, J.M. (1984). *In vivo* mutagenesis by O$^6$-methylguanine built into a unique site in a viral genome. *Proc. Natl Acad. Sci. USA*, **81**, 6271.

LUDLUM, D.B., MEHTA, J.R. & TONG, W.P. (1986). Prevention of 1-(3-deoxycytidyl), 2-(l-deoxyguanosinyl)ethane cross-link formation in DNA by rat liver O$^6$-alkylguanine-DNA alkyltransferase. *Cancer Res.*, **46**, 3353.

McCARTHY, T.V., KARRAN, P. & LINDAHL, T. (1984). Inducible repair of O-alkylated DNA pyrimidines in *E. coli*. *EMBO J.*, **3**, 545.

McCARTHY, T.V. & LINDAHL, T. (1985). Methyl phosphotriesters in alkylated DNA are repaired by the Ada regulatory protein of *E. coli*. *Nucleic Acids Res.*, **13**, 2683.

MORGAN, R.W., CHRISTMAN, M.F., JACOBSON, F.S., STORZ, G. & AMES, B.N. (1986). Hydrogen peroxide-inducible proteins in *Salmonella typhimurium* overlap with heat shock and other stress proteins. *Proc. Natl Acad. Sci. USA*, **83**, 8059.

MYRNES, B., GIERCKSKY, K.E. & KROKAN, H. (1983). Interindividual variation in the activity of O$^6$-methylguanine-DNA methyltransferase and uracil-DNA glycosylase in human organs. *Carcinogenesis*, **4**, 1565.

NAKABEPPU, Y., KONDO, H., KAWABATA, S.-I., IWANAGA, S. & SEKIGUCHI, M. (1985). Purification and structure of the intact Ada regulatory protein of *E. coli* K12, $O^6$-methylguanine-DNA methyltransferase. *J. Biol. Chem.*, **260**, 7281.

NAKABEPPU, Y. & SEKIGUCHI, M. (1986). Regulatory mechanism for induction of synthesis of repair enzymes in response to alkylating agents; Ada protein acts as a transcriptional regulator. *Proc. Natl Acad. Sci. USA*, **83**, 6297.

OLSSON, M. & LINDAHL, T. (1980). Repair of alkylated DNA in *E. coli*: methyl group transfer from $O^6$-methylguanine to a protein cysteine residue. *J. Biol. Chem.*, **255**, 10569.

PAINTER, R.B., & YOUNG, B.R. (1980). Radiosensitivity in ataxia-telangiectasia: a new explanation. *Proc. Natl Acad. Sci. USA*, **77**, 7315.

PEGG, A.E., WIEST, L., FOOTE, R.S., MITRA, S. & PERRY, W. (1983). Purification and properties of $O^6$-methylguanine-DNA transmethylase from rat liver. *J. Biol. Chem.*, **258**, 2327.

ROBINS, P. & CAIRNS, J. (1979). Quantitation of the adaptive response to alkylating agents. *Nature*, **280**, 74.

ROBINS, P., HARRIS, A., GOLDSMITH, I. & LINDAHL, T. (1983). Cross-linking of DNA induced by chloroethyl-nitrosourea is prevented by $O^6$-methylguanine-DNA methyltransferase. *Nucleic Acids Res.*, **11**, 7743.

SAMSON, L. & CAIRNS, J. (1977). A new pathway for DNA repair in *E. coli. Nature*, **267**, 281.

SAMSON, L., DERFLER, B. & WALDSTEIN, E.A. (1986). Suppression of human DNA alkylation – repair defects by *E. coli* DNA-repair genes. *Proc. Natl Acad. Sci. USA*, **83**, 5607.

SEDGWICK, B. (1983). Molecular cloning of a gene which regulates the adaptive response to alkylating agents in *E. coli. Mol. Gen. Genet.*, **191**, 466.

SEDGWICK, B. (1987). Molecular signal for induction of the adaptive response to alkylation damage in *E. coli. J. Cell Sci.*, Suppl. **6**, 215.

SHILOH, Y., TABOR, E. & BECKER, Y. (1982). The response of ataxia-telangiectasia homozygous and heterozygous skin fibroblasts to neocarzinostatin. *Carcinogenesis*, **3**, 815.

SKLAR, R. & STRAUSS, B. (1981). Removal of $O^6$-methylguanine from DNA of normal and xeroderma pigmentosum-derived lymphoblastoid cells. *Nature*, **289**, 417.

TEICHER, B.A., CUCCHI, C.A., LEE, J.B., FLATOW, J.L., ROSOWSKY, A. & FREI, E. (1986). Alkylating agents: *In vitro* studies of cross-resistance patterns in human cell lines. *Cancer Res.*, **46**, 4379.

TEO, I., SEDGWICK, B., DEMPLE, B., LI, B. & LINDAHL, T. (1984). Induction of resistance to alkylating agents in *E. coli*: the *ada* gene product serves both as a regulatory protein and as an enzyme for repair of mutagenic damage. *EMBO J.*, **3**, 2151.

TEO, I., SEDGWICK, B., KILPATRICK, M.W., McCARTHY, T.V. & LINDAHL, T. (1986). The intracellular signal for induction of resistance to alkylating agents in *E. coli. Cell*, **45**, 315.

VAN HOUTEN, B., GAMPER, H., HEARST, J.E. & SANCAR, A. (1986). Construction of DNA substrates modified with psoralen at a unique site and study of the action mechanism of ABC excinuclease on uniformly modified substrates. *J. Biol. Chem.*, **261**, 14135.

WILLIS, A.E. & LINDAHL, T. (1987). DNA ligase I deficiency in Bloom's syndrome. *Nature*, **325**, 355.

YAROSH, D.B. (1985). The role of $O^6$-methylguanine-DNA methyltransferase in cell survival, mutagenesis and carcinogenesis. *Mutation Res.*, **145**, 1.

YAROSH, D.B., FOOTE, R.S., MITRA, S. & DAY, R.S. (1983). Repair of $O^6$-methylguanine in DNA by demethylation is lacking in $Mer^-$ human tumor cell strains. *Carcinogenesis*, **4**, 199.

YAROSH, D.B., RICE, M. & DAY, R.S. (1984). $O^6$-Methylguanine-DNA methyltransferase in human cells. *Mutation Res.*, **131**, 27.

ZARBL, M., SUKUMAR, S., ARTHUR, A.V., MARTIN-ZANCA, D. & BARBACID, M. (1985). Direct mutagenesis of Ha-ras-1 oncogenes by N-nitroso-N-methylurea during initiation of mammary carcinogenesis in rats. *Nature*, **315**, 382.

ZLOTOGORSKI, C. & ERICKSON, L.C. (1983). Pretreatment of normal human fibroblasts and human colon carcinoma cells with MNNG allows chloroethylnitrosourea to produce DNA interstrand crosslinks not observed in cells treated with chloroethylnitrosourea alone. *Carcinogenesis*, **4**, 759.